# Lack of Effects of Extended Sessions of Transcranial Direct Current Stimulation (tDCS) Over Dorsolateral Prefrontal Cortex on Craving and Relapses in Crack-Cocaine Users

**DOI:** 10.3389/fphar.2018.01198

**Published:** 2018-10-23

**Authors:** Jaisa Klauss, Quézia Silva Anders, Luna Vasconcelos Felippe, Leonardo Villaverde Buback Ferreira, Mateus Amorim Cruz, Michael Andreas Nitsche, Ester Miyuki Nakamura-Palacios

**Affiliations:** ^1^Laboratory of Cognitive Sciences and Neuropsychopharmacology, Program of Post-Graduation in Physiological Sciences, Health Sciences Center, Federal University of Espírito Santo, Vitória, Brazil; ^2^Leibniz Research Centre for Working Environment and Human Factors, Dortmund, Germany; ^3^Department of Neurology, University Medical Hospital Bergmannsheil, Bochum, Germany

**Keywords:** crack-cocaine use disorder, tDCS, dorsolateral prefrontal cortex, craving, relapses

## Abstract

**Background:** Non-invasive brain stimulation such as transcranial direct current stimulation (tDCS) has been investigated as additional therapeutic tool for drug use disorder. In a previous study, we showed that five sessions of tDCS applied bilaterally over the dorsolateral prefrontal cortex (dlPFC) reduced craving to the use of crack-cocaine in inpatients from a specialized clinic. In the present study, we examine if an extended number of sessions of the same intervention would reduce craving even further and affect also relapses to crack-cocaine use.

**Methods:** A randomized, double-blind, sham-controlled, clinical trial with parallel arms was conducted (https://clinicaltrials.gov/ct2/show/NCT02091167). Crack-cocaine patients from two private and one public clinics for treatment of drug use disorder were randomly allocated to two groups: real tDCS (5 cm × 7 cm, 2 mA, for 20 min, cathodal over the left dlPFC and anodal over the right dlPFC, *n* = 19) and sham-tDCS (*n* = 16). Real or sham-tDCS was applied once a day, every other day, in a total of 10 sessions. Craving was monitored by a 5-item obsessive compulsive drinking scale once a week (one time before, three times during and once after brain stimulation) over about 5 weeks and relapse was monitored after their discharge from clinics for up to 60 days.

**Results:** Craving scores progressively decreased over five measurements in both sham- and real tDCS groups. Corrected Hedges’ within-group (initial and final) effect sizes of craving scores were of 0.77 for the sham-tDCS and of 0.97 for the real tDCS group. The between-groups effect size was of 0.34, in favor of the real tDCS group over sham-tDCS group. Relapse rates were high and quite similar between groups in the 30- and 60-days follow-up after discharge from the hospital.

**Conclusion:** Extended repetitive bilateral tDCS over the dlPFC had no add-on effects over regular treatment when considering craving and relapses to the crack-cocaine use in a sample of crack-cocaine patients with severe use disorder. Different tDCS montages targeting other cortical regions and perhaps additional extension of sessions need to be investigated to reach more efficiency in managing craving and relapses to crack-cocaine use.

## Introduction

Cocaine is a highly addictive substance consumed by more than 17 million people worldwide (United Nations, 2017), either as a cocaine hydrochloride salt – usually snorted or diluted in water and injected, or as a “crack” cocaine base – frequently smoked due to its lower melting temperature (Hatsukami and Fischman, 1996). Along with amphetamine-type substances with whom it shares similar pharmacological mechanisms, cocaine is a “stimulant” drug (Rothman et al., 2001), increasing dopaminergic activity due to a blockage of dopamine reuptake pumps in the presynaptic membrane and thus enhancing the activation of the mesocorticolimbic dopamine reward circuitry, a critical mechanism in causing its behavioral effects (Dackis and O’Brien, 2001; Howell, 2008).

Cocaine use disorder is a chronic relapsing disease characterized by repetitive and compulsive drug-seeking behavior and drug abuse despite negative consequences (Goldstein and Volkow, 2002; Karila et al., 2012) with craving being recently described as an essential feature of the disease (DSM-5, 2013). Craving is defined as an intense desire or urge for the drug that may occur at any time but might be triggered by environmental features previously associated with drug use (DSM-5, 2013).

The prefrontal cortex (PFC) plays a major role in cognition (Miller and Cohen, 2001), being responsible for functions such as working memory (D’Esposito et al., 2000; D’Esposito and Postle, 2015), learning, planning tasks and reasoning, which are relevant for balancing environmental exploration and regulation of behavior (Koechlin, 2016), including modulatory aspects of motivation, emotions and behavior (Caballero et al., 2016). Long-term crack-cocaine exposure has been associated to both, decreased gray matter volume in cortical regions (Franklin et al., 2002), including prefrontal areas, and decreased cognitive performance (Meyer et al., 2014). These effects are added by a reduction of neurotransmitters and molecular neural activation markers (Baltazar et al., 2013). PFC dysfunction has been directly related to drug use disorders (DUD) (Kalivas, 2008; Goldstein and Volkow, 2011; Nakamura-Palacios et al., 2016; Vaquero et al., 2017), especially to their negative outcomes such as high relapse rates and craving (Goldstein and Volkow, 2011).

Transcranial direct current stimulation (tDCS) is a non-invasive brain stimulation tool which has proven to be efficient in modulating brain activity (George and Aston-Jones, 2010). Previous studies already demonstrated favorable effects of tDCS compared to sham stimulation in substance use disorders and craving. Accordingly, we have demonstrated that in crack-cocaine dependent subjects, five sessions of right anode/left cathode bilateral stimulation of the dlPFC was able to significantly reduce craving both during and after treatment in the real tDCS group as compared to a sham-tDCS group (Batista et al., 2015). Thus, we hypothesized that extension of repetitive bilateral dlPFC tDCS to ten sessions would have a more pronounced effect on craving in crack-cocaine substance use disorder, considering that a successful management of craving during treatment is highly desirable to prevent dropouts and relapses.

## Materials and Methods

We report this clinical trial according to CONSORT guidelines. This trial was registered under Clinical Trials.gov number NCT02091167.

### Participants

All subjects were informed about the purposes of the experiment by the principal investigator and signed a written consent before entering the study.

Thirty-five patients, 29 men and 6 women, who met DSM V criteria for cocaine (crack) use disorder were recruited between June of 2015 and April of 2018 from three specialized clinics for DUD treatment (one public and two private hospitals) from Espírito Santo State, Brazil. They all received standard treatment given by the clinics, consisting of psychosocial approaches – conducted by a professional team of psychologists, nurses, social workers and physicians – sometimes combined with adjunctive pharmacotherapy including benzodiazepines, B-complex vitamins, disulfiram and, if necessary, antidepressants, anxiolytics, antipsychotics, antihypertensive and gastric medication. It must be mentioned that in the public hospital, from where half of the patients was recruited, they were not allowed to have any medication, except non-opioid pain relievers when absolutely necessary, after they had been admitted to the hospital. Therefore, half of the patients were free of medication during the sham- or DC-stimulation. From the other half patients coming from the two private clinics, few of them were medicated (antipsychotics, antidepressants or mood stabilizers) during brain stimulation procedures.

**FIGURE 1:**
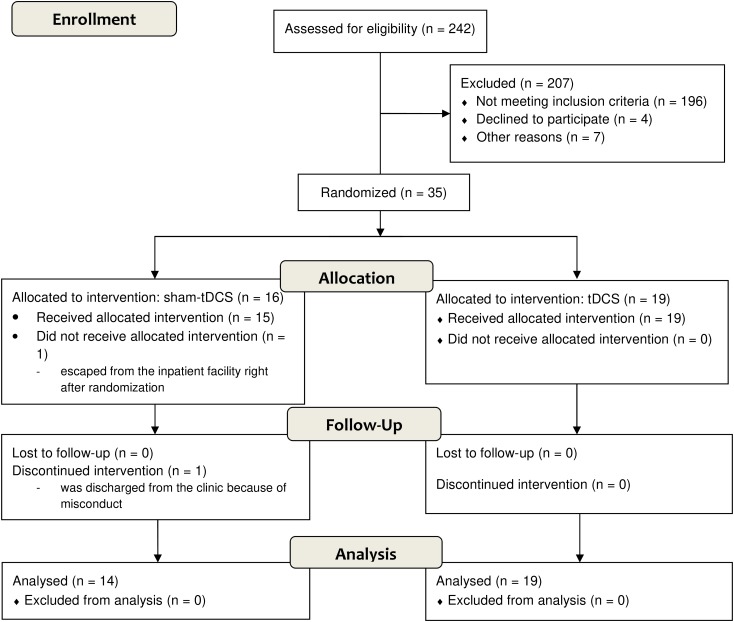
Flow diagram according to CONSORT 2010.

There were two dropouts in the sham-tDCS group that were excluded after randomization (Figure 1). One patient escaped from the treatment facility and the other had to be discontinued because of precocious discharge from the clinic for misconduct.

The inclusion criteria for this study were: (1) male and female patients over the age of 18 years; (2) met criteria for crack-cocaine use disorder according to the ICD-10 Classification of Mental and Behavioral Disorders and the Diagnostic and Statistical Manual of Mental Disorders, fifth edition, as determined by clinical evaluation; (3) in stable clinical condition with no need for emergency care; (4) able to read, write, and speak Portuguese; and (5) no severe withdrawal signs or symptoms at baseline.

Furthermore, exclusion criteria included: (1) a condition of intoxication or withdrawal due to a substance other than crack-cocaine, (2) unstable mental or medical disorder or substance abuse or addiction other than crack-cocaine use disorder, except nicotine and/or caffeine; (3) diagnosis of epilepsy, convulsions, or delirium tremens during abstinence from crack-cocaine; (4) a previous history of drug hypersensitivity or adverse reactions to diazepam or other benzodiazepines and haloperidol; (5) any contraindication for electrical brain stimulation procedures such as electronic implants or metal implants.

The study was approved by the Brazilian Institutional Review Board of the Federal University of Espírito Santo (CAAE 19403713.6.0000.5060), Brazil, and all patients signed a written informed consent form. The study was conducted in strict adherence to the Declaration of Helsinki and is in accordance with the ethical standards of the Committee on Human Experimentation of the Federal University of Espírito Santo, ES, Brazil.

### DC Stimulation

A randomized double-blind clinical trial tDCS protocol was used in the study. Stimulation was done using a DC stimulator (DC-Stimulator Plus, NeuroConn, Ilmenau, Germany) with two carbonated silicone electrodes (35 cm^2^) with a thick layer of high-conductive EEG gel beneath them according to our previous study (Nakamura-Palacios et al., 2012). Electrodes were placed based on the international 10–20 electrode placement system. For tDCS, the cathode was placed over the left dlPFC (F3) while the anode was placed over the right dlPFC (F4). Each session of tDCS lasted for 20 min with fade-in and fade-out periods of 30 s each. Intensity was set to 2 mA. For sham tDCS, the electrodes were placed at the same positions.

During active tDCS treatment, subjects typically reported tingling sensations under the electrodes area, which rapidly faded (Batista et al., 2015). Our sham intervention was therefore designed to provide an initial period of tingling - the stimulator was automatically switched off after 30 s of either anodal or cathodal stimulation – so that similar sensations are perceived during active and sham tDCS protocols, thus serving as an ideal control condition (Nitsche et al., 2003; Gandiga et al., 2006; Batista et al., 2015). Data and instructions in the device display are identical in active and sham settings.

For the sham stimulation procedure, the stimulator automatically switched off after 30 s of either anodal or cathodal stimulation yielding sensations typically elicited by tDCS.

### Craving Assessment

Craving was scored through a brief scale composed of five items (1, 2, 4, 5, and 13) of the Obsessive-Compulsive Cocaine Use Scale, also known as the Obsessive–Compulsive Cocaine Scale (OCCS), which are based on the Obsessive-Compulsive Drinking Scale (Anton et al., 1995, 1996; Anton, 2000), as proposed by Hormes et al. (2012) and Vorspan et al. (2012). These five-item scales assess craving in a narrow sense according to de Wildt et al. (2005).

Through this brief scale it is possible to quantify thoughts and feelings (obsessions), and behavioral intentions (de Wildt et al., 2005), answered on a scale ranging from 0 to 4, resulting in a total score between 0 and 20. Patients are questioned on how much of the time (total per day), when the drug is not used, is occupied by thoughts, ideas, desires, or impulses related to crack-cocaine and its effects; how frequently these thoughts, ideas, desires, or impulses related to crack-cocaine and its effects occur; how much distress or disturbance these ideas, thoughts, impulses or desire related to crack-cocaine use cause when the person is under withdrawal; how much effort they have to make to resist these thoughts, ideas, desires, or impulses, or how much energy they have to spend to think of something else when they enter the mind under withdrawal; and finally ask about their drive to use crack-cocaine.

The OCCS was applied in the week before the beginning of the real or sham-tDCS treatment, during the treatment (second, third, and fourth weeks) and in the week after the end of the brain stimulation application, resulting in five time-points measurements.

### Relapses in 30- and 60-Days Follow-Up

After their discharge from the hospital, patients from sham- and real tDCS groups were followed-up for at least 60 days regarding crack-cocaine use relapses. A use relapse was defined as the first episode of return to the previous uncontrolled pattern of crack-cocaine use (rocks per day) (Klauss et al., 2014). Information about relapse were gathered directly when patients regularly returned to the hospital for clinical follow-up after their discharge and/or by self-report or reports of family members by telephone calls.

### Procedures

**FIGURE 2:**
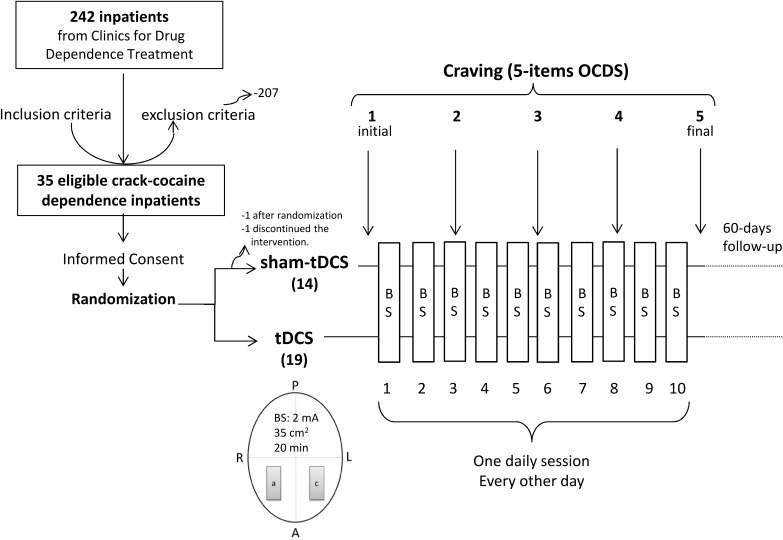
Diagram of the general procedure: eligible crack-cocaine users were recruited from clinics for treatment of drug use disorder, signed the informed consent form and were randomized to receive repetitive bilateral (cathode left/anode right over the Dorsolateral Prefrontal Cortex) transcranial Direct Current Stimulation (2 mA, 35 cm^2^, stimulation for 20 min) every other day in 10 sessions. Craving to the use of crack-cocaine was examined by 5 items from the Obsessive–Compulsive Cocaine Use Scale once a week for 5 weeks (the week before treatment, during the second, third and fourth treatment weeks, and the week after treatment). A, anterior; P, posterior; R, right; L, left; a, anode; c, cathode; BS, brain stimulation.

Those patients who were eligible according to inclusion and exclusion criteria described above and agreed to participate in this study signed an informed consent sheet (Figure 2). All data were originally acquired from participants entering this single research center clinical trial to investigate the efficacy of tDCS treatment.

After global physical and clinical examination subjects were randomly assigned to one of the two groups (sham- and real tDCS) in a 1:1 ratio using a computer-generated block randomization sequence that was kept with the unblinded study coordinator (not involved in the recruitment). The co-investigator conducting treatments was only given a list of 5-number blinding codes to be loaded to the DC-stimulator before each session of brain stimulation. The device is previously settled with specific settings for the study.

After patients had been admitted to the hospitals, they were maintained under regular treatment for 30 days in average or until they had reached a global clinical stabilization, to have them started in the sham- or real tDCS treatments. The brain stimulation application was then performed in one 20-min session a day, every other day, including weekends, up to a total of 10 sessions, always in the afternoon period, in the following 5 weeks and they were followed-up after the end of the stimulation treatment for up 60 days after their discharge from the hospitals (Figure 2).

Craving was measured once a week over 5 weeks (once before the beginning of the stimulation sessions, three times over the stimulation sessions and once more after the end of stimulation sessions) with a total of five time-points measurements (Figure 2). Relapses were collected after discharge from the hospital up to 60 days after intervention.

Participants and experimenters were blinded for brain stimulation assignments from the beginning of the study protocol up to the end of the 60-days follow-up after the end of sham- or real tDCS treatment, resulting in a double-blind experimental design.

### Statistical Analysis

We powered the study for a medium effect size based on the results of our previous study (Batista et al., 2015) in which the effect size (partial η_2_) for the main within-subject factor in the respective two-way ANOVA with repeated measures was 0.10384 when comparing craving scores once before, twice during and once after 5-sessions of tDCS (four time-points). The tDCS electrode montage was identical in the studies, and patient populations of crack-cocaine users were similar. We used the correction for SPSS input into the G^∗^Power 3.1.9.2. With this effect size, for the two-way mixed model ANOVA of the present study with the within factor craving measurements, the between-subject factor tDCS condition, the dependent variable craving score, and craving measurements x condition interaction as the primary outcome parameter, with a power of 80%, and a two-sided probability of a type I error of 5%, the resulting minimum sample size was 30 participants. To account for waiving or dropouts, which were expected to be very common in this condition, we increased the estimated sample size to approximately 10%, resulting in 33 subjects in total (approximately 16–17 subjects in each group).

Most of data (age, patterns of crack-cocaine use, 5-items OCCS) were normally distributed according to the D’Agostino and Pearson normality test, thus they were analyzed by parametric tests. Between-group (sham- and real tDCS) comparisons were conducted by unpaired *Student’s t*-tests. For all other non-parametric data (gender, schooling, employment, marital state, and tobacco use), Chi-square or Fisher tests were used to compare sham and real tDCS groups.

Besides the two-way ANOVA with repeated measures followed by Bonferroni-corrected *t*-tests, linear regression analyses were done over craving scores obtained along the 4-week treatment (five time-points measurements) for both groups. Additional comparisons between initial and final OCDS scores were done by paired *t*-tests for each group, and differences between final and initial scores were compared between sham-tDCS and real tDCS groups with unpaired *t*-test. Effect sizes were calculated using Cohen’s d and corrected by Hedges’s *g_s_* for unpaired and Hedges’ *g_av_* for paired *t*-tests (Lakens, 2013).

A two-tailed *p*-value of 0.05 or less was considered to indicate statistical significance. SPSS Statistics Base 24.0 (SPSS Inc., United States) and GraphPad Prism 7.0 (GraphPad Software Inc., United States) were employed for statistical analysis and graphic presentations.

## Results

### Baseline Data

Baseline socio-demographic characteristics and patterns of drug use are presented in Tables 1, 2.

**Table 1:** Socio-demographic characteristics of the total sample of crack-cocaine users (*n* = 33), subdivided in subjects submitted to bilateral repetitive transcranial Direct Current Stimulation (real tDCS: cathode left/anode right dorsolateral Prefrontal Cortex, 2 mA, 35 cm^2^, 20 min, 10 sessions, every other day, *n* = 19) or placebo intervention (sham-tDCS: *n* = 14).

	Crack-cocaine users (*n* = 33)		Groups			
			Sham-tDCS (*n* = 14)	Real tDCS (*n* = 19)		*p-value*
Age *[mean (SD)]*		35.03 (8.7)	35.0 (9.6)	35.1 (8.2)	*t(31) = -0.02*	*0.99*
Gender *n* (*%*)	Male	27 (81.8 %)	12 (85.7 %)	15 (78.9%)	*Fisher = 1.0*	*0.49*
	Female	6 (18.2%)	2 (14.3%)	4 (21.1%)		
Years of education *n* (*%*)	Between 6 and 9	11 (33.3%) (9M:2F)	4 (28.6%) (3M:1F)	7 (36.8%) (6M:1F)	*X_2_ T = 0.28*	*0.87*
	Between 10 and 13	15 (45.5%) (12M:3F)	7 (50.0%) (6M:1F)	8 (42.1%) (6M:2F)	*X_2_ M = 0.68*	*0.69*
	Above 13	7 (21.2%) (6M:1F)	3 (41.4%) (3M:0F)	4 (21.1%) (3M:1F)	*X_2_ F = 0.70*	*0.92*
Employment situation *n* (*%*)	Formal job	6 (18.2%) (5M:1F)	4 (28.6%) (4M:0F)	2 (10.5%) (1M:1F)	*X_2_ T = 4.57*	*0.21*
	Unemployed	17 (51.5%) (14M:3F)	8 (57.1%) (6M:2F)	9 (47.4%) (8M:1F)	*X_2_ M = 4.47*	*0.22*
	Freelance	8 (24.2%) (6M:2F)	1 (7.1%) (1M:0F)	7 (36.8%) (5M:2F)	*X_2_ F = 3.0*	*0.22*
	Retired	2 (6.1%) (2M:0F)	1 (7.1%) (1M:0F)	1 (5.3%) (1M:0F)		
Marital state *n* (*%*)	Single	22 (66.7%) (17M:5F)	10 (71.4%) (9M:1F)	12 (63.2%) (8M:4F)	*X_2_ T = 4.67*	*0.32*
	Married	2 (6.1%) (2M:0F)	0 (0.0%) (0M:0F)	2 (10.5%) (2M:0F)	*X_2_ M = 3.91*	*0.27*
	Common-law	5 (15.2%) (5M:0F)	1 (7.1%) (1M:0F)	0 (0.0%) (0M:0F)	*X_2_ F = 2.40*	*0.12*
	Divorced	3 (9.1%) (3M:0F)	2 (14.3%) (2M:0F)	1 (5.3%) (1M:0F)		
	Widow	1 (3.0%) (0M:1F)	1 (7.1%) (0M:1F)	0 (0.0%) (0M:0F)		
Tobacco use *n (%)*	Yes	22 (66.7%) (19M:3F)	11 (78.6%) (10M:1F)	11 (57.9%) (9M:2F)	*Fisher T = 0.28*	*0.19*
	No	11 (33.3%) (8M:3F)	3 (21.4%) (2M:1F)	8 (42.1%) (6M:2F)	*Fisher M = 0.24*	*0.19*
					*Fisher F = 1.0*	*0.80*


*T, total; M, male; F, female.*

**Table 2:** Patterns of crack-cocaine use, impression of real-/placebo intervention and confidence of this impression, and adverse events, for the total sample of crack-cocaine users (*n* = 33), and subdivided groups in subjects submitted to bilateral repetitive transcranial Direct Current Stimulation (real tDCS: cathode left/anode right dorsolateral Prefrontal Cortex, 2 mA, 35 cm^2^, 20 min, 10 sessions, every other day, *n* = 19) or placebo intervention (sham-tDCS: *n* = 14).

	Crack-cocaine users (*n* = 33)			Groups			
				Sham-tDCS (*n* = 14)	Real tDCS (*n* = 19)		*p*-value
**Crack-cocaine use**							
Age at onset of crack-cocaine use *[mean (SD)]*			23.6 (8.1)	24.4 (9.6)	23.0 (7.0)	*t(31) = 0.49*	*0.63*
Amount of crack-cocaine used^#1^ (rocks/day) *[mean (SD)]*			19.1 (21.5)	19.5 (26.8)	18.7 (17.2)	*t(30) = 0.10*	*0.92*
Days of abstinence before study *[mean (SD)]*			32.8 (15.5)	33.7 (17.5)	32.2 (14.2)	*t(31) = 0.28*	*0.78*
**Impression *n (%)***							
sham (placebo)			1 (3.0%)	0 (0.0%)	1 (5.3%)		
tDCS treatment			32 (97.0%)	14 (100.0%)	18 (94.7%)		
Confidence in their	(1) None		0 (0.0%)	0 (0.0%)	0 (0.0%)	*X_2_ = 1.23*	*0.75*
impression	(2) Little		2 (6.1%)	1 (7.1%)	1 (5.3%)		
	(3) Medium		5 (15.2%)	2 (14.3%)	3 (15.8%)		
*n (%)*	(4) Very confident		15 (45.5%)	5 (35.7%)	10 (52.6%)		
	(5) Extremely confident		11 (33.3%)	6 (42.9%)	5 (26.3%)		
**Adverse events *n (%)^#2^***							
None			9 (27.3%)	6 (42.9%)	3 (15.8%)	*X_2_ = 5.71*	*0.22*
Tingling in the scalp			21 (63.6%)	7 (50.0%)	14 (73.7%)		
Headache			1 (3.0%)	1 (7.1%)	0 (0.0%)		
Drowsiness			1 (3.0%)	0 (0.0%)	1 (5.3%)		
Burning sensation of the scalp			1 (3.0%)	0 (0.0%)	1 (5.3%)		


*^#1^One patient from tDCS group was not included in this analysis because it has been reported the use of 1000 rocks/day. ^*#2*^No other adverse event asked was registered (neck and scalp pain, itching, skin redness, acute mood changes, trouble concentrating)*.

Crack-cocaine users were young aged, with an average age of 35 years in the total sample, the majority was male (82%), had a good educational degree (45.5% of them had 10 to 13 years and 21.2% above 13 years of education), was unemployed (51.5%), and single (66.7%) (Table 1). In addition, the majority of the participants (66.7%) were tobacco smokers (Table 1). No socio-demographic parameter differed between sham-tDCS and real tDCS groups (Table 1).

Participants started to use crack-cocaine on average at 23.6 years of age, consumed on average 19.1 rocks per day (some of them used 80–100 rocks per day, one reported the use of 1000 rocks per day), and they were about 33 days abstinent before the start of the experimental protocol (Table 2). None of these characteristics differed between sham and real tDCS groups (Table 2).

Patients were kept in a restrictive environment for drug use during the treatment. They were blinded for tDCS treatment. When they were questioned at the end of the treatment about their impression of what treatment they received, 32 (97%) subjects answered that they had received real tDCS (Table 2). All subjects from the sham-tDCS group (100%) and almost all subjects (94.7%) from the real tDCS group answered that they had received real tDCS treatment. The great majority, 26 from the total (78.8%), including 11 subjects (78.6%) from the sham-tDCS group and 15 participants (78.9%) from the real tDCS group, were very to extremely confident regarding their impression of the treatment condition. There were no statistically significant differences between groups for both parameters, impression and confidence (Table 2).

### Adverse Events

After treatment, subjects were asked regarding adverse events such as headache, neck and scalp pain, tingling, itching, skin redness, burning sensation of the scalp, sleepiness, acute mood changes, trouble concentrating, and others. From these potential events, a tingling sensation was the most frequent event reported by 21 subjects (63.6%) in the total sample, 7 (50%) from the sham-tDCS and 14 (73.7%) from the real tDCS group (Table 2). Six subjects (42.9%) from the sham-tDCS group and three from the real tDCS group (15.8%) reported no events at all. Headache was reported by one subject from the sham-tDCS group (7.1%), drowsiness (5.3%) and burning sensation of the scalp (5.3%) by one subject each from the real tDCS group. No other adverse events were reported by crack-cocaine patients from both groups in this study and no significant difference was found between groups (Table 2).

### Craving: 5-Items OCCS

**FIGURE 3:**
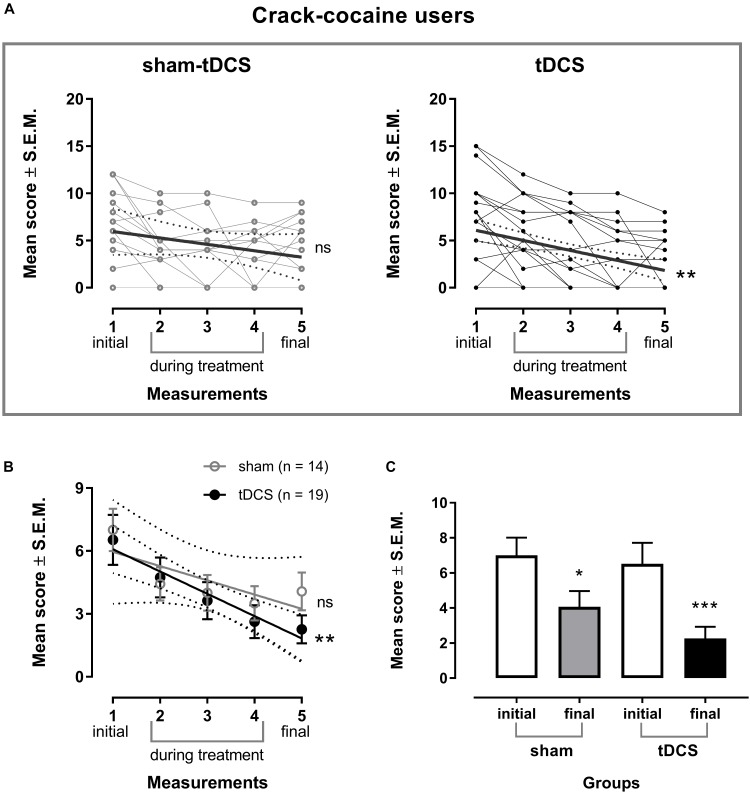
Craving is shown individually **(A)** and as the mean of the 5 item score from Obsessive-Compulsive Cocaine Use Scale score ± standard error of means (SEM) **(B)** in the week before treatment (1 initial), the second (2), third (3) and fourth (4) weeks during the treatment, and the week after treatment (5 final) with bilateral repetitive transcranial Direct Current Stimulation (tDCS, 2 mA, 35 cm^2^: cathode left/anode right over the Dorsolateral Prefrontal Cortex; stimulation for 20 min every other day of 10 sessions; *n* = 19) or placebo (sham-tDCS; *n* = 14) in crack cocaine users. Linear regression of the real tDCS group: ^∗∗^*p* < 0.01. **(C)** Mean scores of craving shown in the week before and the week after treatment in the sham-tDCS and real tDCS groups. ^∗^*p* < 0.05 and ^∗∗∗^*p* < 0.001 when compared to initial scores (paired *t*-test).

A two-way mixed model ANOVA examining the intervention effect on craving showed that both, real and sham-tDCS groups differed in craving scores over time (Figure 3). There was a significant within-subject main effect of craving. We applied the Greenhouse–Geisser method to correct the degrees of freedom, as sphericity (Mauchley test) could not be assumed [*F*(2.6,81.8) = 15.21, *p* < 0.000001, $\eta_{p}^{2}$ = 0.33, $\eta_{G}^{2}$ = 0.122]. No significant interaction was found between groups and five time-points craving measurements, and there were no significant between-subjects’ effects. Bonferroni’s multiple comparisons tests showed that craving scores were significantly smaller in the 2nd, 3rd, 4th, and 5th time-points when compared to the 1st time-point (adjusted *p*-value < 0.05, <0.01, <0.001 and <0.01, respectively) in the sham-tDCS group. In the real tDCS group, craving scores were smaller in the 3rd, 4th, and 5th time-points when compared to the 1st time-point (adjusted *p*-value < 0.001, <0.0001, <0.0001, respectively), and in the 4th and 5th time-points when compared to the 2nd time-point (*p* < 0.05 and <0.01).

As shown by the respective regression analysis, craving scores decreased linearly from baseline (week before treatment) to the week after treatment in the real tDCS group only [*Y* = 7.147 – 1.063X, *r*^2^ = 0.9466, *F*(1,3) = 53.13, *p* = 0.0053] (Figure 3B).

When comparing craving scores obtained before (initial) and after (final) treatment by paired *t*-tests, statistically significant differences were observed for the sham-tDCS group [*t*(13) = 2.86, *p* = 0.0134, 95% CI [-5.141, -0.716]] and the real tDCS group [*t*(18) = 4.77, *p* = 0.0002, 95% CI [-6.143, -2.384]] (Figure 3C), showing that craving scores were significantly smaller than baseline values after 10 sessions in both groups. The corrected effect size for the paired *t*-tests between initial and final craving scores of the sham-tDCS group by Hedges’s *g_av_* was 0.77 (initial mean score = 7.0, *SD* = 3.78; final mean score = 4.1, *SD* = 3.36), and of the real tDCS group it was 0.97 (initial mean score = 6.5, *SD* = 5.2; final mean score = 2.3, *SD* = 2.90). The effect size calculated indicates that after controlling for individual differences, the likelihood that craving scores of a crack-cocaine patient under sham-tDCS treatment are lower for the final than for initial mean score is 77% and under real-tDCS treatment is 84%.

When comparing the mean change scores contrasting data obtained after 10 sessions (final) versus baseline (initial) between groups (sham-tDCS vs. tDCS), the between groups comparison was not statistically significant, and the corrected effect size by Hedges’s *g_s_* for two independent samples was 0.34 (mean difference of sham-tDCS = -2.93, *SD* = 3.83; mean difference of real tDCS = -4.26, *SD* = 3.90). The effect size indicates that the chance for a randomly selected pair of subjects, the probability of a lower score of a crack-cocaine patient from the real tDCS-group, as compared to the score of a crack-cocaine patient from the sham-tDCS group, is 60%.

### Crack-Cocaine Use Relapses

Four crack-cocaine patients were lost to the follow-up, two from the sham- and two others from the real tDCS groups, most of them because they and/or their relatives could not be reached after many attempts. Unfortunately, two other patients, both from the sham-tDCS group, were deceased after they had been discharged from the hospital and had returned to their usual environment, both because of drug trafficking issues, after they had relapsed to drug use.

**FIGURE 4:**
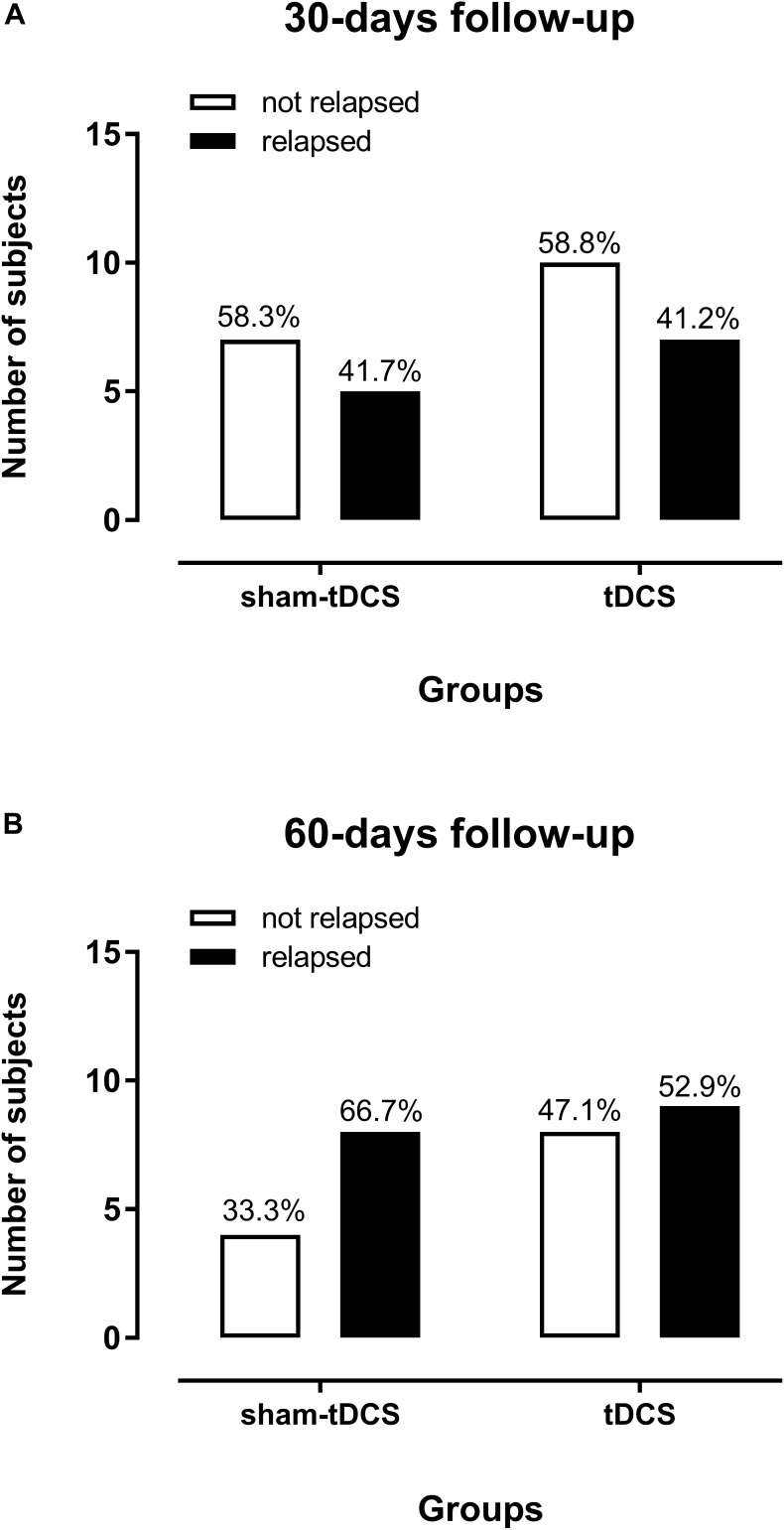
Crack-cocaine use relapses in dependent patients in the 30-days **(A)** and 60-days **(B)** follow-up after ten sessions of sham- (*n* = 12) or real tDCS (*n* = 17) applied over the bilateral dorsolateral Prefrontal Cortex. Two patients from each group were lost to follow-up.

Crack-cocaine use relapses up to 30 days after the end of 10 sessions of brain stimulation were frequent (41.7% in the sham-tDCS group and 41.2% in the tDCS group), with no statistical difference between groups (Figure 4A). When considering the 60-days follow-up (Figure 4B), the frequency of relapses was increased in both groups, 66.7% in the sham-tDCS group and 52.9% in the real tDCS group, with no significant differences between groups. The respective odds-ratio for relapse was 1.02 (95% CI: 0.21 – 4.44) at 30-days follow-up, and 1.78 (95% CI: 0.42 – 6.81) for the comparison of sham-tDCS with real tDCS group 60 days after intervention.

## Discussion

In the present study, craving scores progressively decreased in crack-cocaine users from both intervention groups over the course of 10-sessions application of sham or real tDCS treatment. The craving scores and relapses in crack-cocaine patients treated with ten sessions of bilateral prefrontal tDCS was obtained in comparison to a well-matched placebo (sham) control group, as patients from sham- and real-tDCS groups were equivalent in their socio-demographic characteristics such as age, gender, schooling, employment and marital state conditions and characteristics of crack-cocaine use, considering the amount of daily use, the age at the onset of crack-cocaine use and days of abstinence before tDCS.

These results showed the same direction of those observed in our previous study with five sessions of the same montage of tDCS in crack-cocaine users from the same metropolitan region (Batista et al., 2015), but showing no significant difference on craving score decreasing when compared to placebo control. It must be pointed out that our previous sample was constituted by younger subjects (mean age of 30.4 ± 9.0 SD years, *n* = 36), and in the present study patients was slightly older (mean age of 35 ± 8.7 SD years); the amount of daily crack-cocaine use was smaller in the previous (mean of 13.1 ± 11.3 SD rocks per day), than in the present study (mean of 19.1 ± 21.5 SD rocks per day); and the mean craving score at baseline was lower in the previous (3.6 ± 3.8 SD) than that in the present sample (6.7 ± 4.6 SD). The respective comparison of these mean craving scores between samples of the previous and the present studies was statistically significant, [*t*(67) = 3.063, *p* = 0.0032, unpaired *t*-test], indicating that crack-cocaine users in the present study exhibited a more severe pattern of drug use and higher craving scores at the beginning of the study.

However, at the end of five sessions of tDCS in the previous study and of 10 sessions of tDCS in the present study, craving scores were comparable between both samples (1.8 ± 2.04 SD after 5 sessions, 2.3 ± 2.9 SD after 10 sessions). Because the mean craving score was larger at baseline in the present study, there was a larger difference between initial to final scores and consequently the effect size was larger in the present study. The calculated Hedges *g*_av_ was 0.54, corresponding to a medium effect size according to Cohen’s convention (Cohen, 1992) in our previous study, and Hedges *g*_av_ was 0.97, indicating a large effect size in the present study, a 1.8-fold increase in the effect size after 10 sessions of tDCS. Therefore, although the final mean scores indicate residual craving after 5 and 10 sessions with no statistically significant difference between mean final craving scores, the reduction was larger in the present study, which is important when considering the more severe profile of crack-cocaine users included. It must be considered that in the present study the effect size was also high in the sham-tDCS group, thus the regular treatment showed to be similarly effective.

However, relapses to the use of crack-cocaine after subjects had been discharged from the hospital were very similar between groups in the first 30 days and almost similar after 60 days. A high proportion from both, the sham-tDCS and real tDCS groups (41.7 and 41.2, respectively) relapsed in the first 30 days after intervention, and a higher proportion of subjects relapsed in both groups up to 60 days after intervention, 66.7% in the sham-tDCS group and 52.9% in the real tDCS group, which, unfortunately, are usually seen in this DUD (Dias et al., 2011; Czermainski et al., 2017).

Besides our previous tDCS study in crack-cocaine users, which was already mentioned above (Batista et al., 2015), non-invasive brain stimulation techniques have shown a modest beneficial effects on craving for other psychostimulants such as cocaine (Camprodon et al., 2007; Gorini et al., 2014; Rapinesi et al., 2016), methamphetamine (Shahbabaie et al., 2014; Shariatirad et al., 2016) and nicotine (Fecteau et al., 2014). However, effects on relapses had not been largely investigated so far.

Some limitations of this study need to be considered. Although the sample size was satisfactory considering the calculation of the effect size in our previous study, it is still small and relevantly restricted by inclusion and exclusion criteria, limiting generalizability of our results. From a very large sample (244 subjects) of interviewed inpatients from DUD treatment clinics, only 35 (approximately 14%) were primordially crack-cocaine users (the majority were polydrug users) showing eligibility to be included and consented to participate in this study. Surrogate parameters obtained additionally in this study, but beyond the scope of the present report, including cognitive performance parameters, clinical outcomes such as anxiety and depression symptoms, quality of life, and electrophysiological data will be processed and reported in subsequent publications to help to understand the extension of neuromodulatory effects of tDCS and mechanisms that may underlie these effects. Finally, it is important to mention that this study explored one specific montage of tDCS application and examined its efficacy based on an extended number of sessions, and although it showed mild effects on craving scores over the period of treatment, it had no significant after effects on relapses to crack-cocaine use after hospital discharge. Therefore, considering the severity of this DUD, it is highly recommended to consider other electrode montages and sizes, a further extension of sessions and different parameters of brain stimulation combined with pharmacological therapeutics and other adjunctive approaches in future studies.

In summary, this study shows that decreasing craving scores in severe crack-cocaine addicted after 10 sessions of bilateral tDCS over the dlPFC (cathodal right and anodal left) was equivalent to regular treatment for crack-cocaine use disorder alone and there were no after effects on relapses after discharge from the hospital. Therefore, this stimulation protocol showed no effective enhancement on the reduction of crack-cocaine craving in this sample of crack-cocaine users. Other tDCS parameters, such as different montages targeting different cortical regions and further extension of sessions need to be investigated to reach more efficiency in managing craving and especially relapses to the crack-cocaine use.

## Author Contributions

EN-P and JK conceived of the presented idea and contributed with important theoretical and technical content. JK coordinated the recruitment of patients, collected clinical, cognitive and electrophysiological data, applied the tDCS and made important theoretical contributions. LVF, LVBF, and MC helped to collect cognitive and electrophysiological data and made important theoretical contributions. EN-P supervised the study, ran the data analysis, and organized the manuscript. MN made important theoretical contributions. All authors discussed the results and contributed to the final manuscript and approved it for publication.

## Conflict of Interest Statement

MN is member of the scientific advisory board of Neuroelectrics. The remaining authors declare that the research was conducted in the absence of any commercial or financial relationships that could be construed as a potential conflict of interest.
